# Amiodarone-Induced Immune Thrombocytopenia: A Rare Hematologic Side Effect of a Common Cardiac Drug

**DOI:** 10.7759/cureus.13671

**Published:** 2021-03-03

**Authors:** Hardik Chhatrala, Shreya Ghetiya, Michael Chahin, Lara Zuberi, Walter Quan

**Affiliations:** 1 Oncology, University of Florida College of Medicine – Jacksonville, Jacksonville, USA; 2 Cardiology, University of Florida College of Medicine – Jacksonville, Jacksonville, USA; 3 Internal Medicine, University of Florida College of Medicine – Jacksonville, Jacksonville, USA

**Keywords:** thrombocytopenia, amiodarone immune thrombocytopenia, drug-induced immune thrombocytopenia (ditp)

## Abstract

Thrombocytopenia is a rare immune-mediated hematologic complication of amiodarone. We describe a case of delayed-onset amiodarone-induced thrombocytopenia in a 72-year-old male and highlight the process of working it up. A timely diagnosis of drug-induced immune thrombocytopenia is crucial in order to minimize unnecessary testing, avoid treatments with potential harm, and prevent life-threatening hemorrhagic complications.

## Introduction

Thrombocytopenia is defined as platelet count lower than 150 K/mm^3^ and is commonly encountered in hospitalized patients [1]. Drug-induced immune thrombocytopenia (DITP) is marked by a severely reduced platelet count, often less than the range of 20 K/mm^3^ where it could be associated with life-threatening bleeding. The underlying mechanism is understood to be immune-mediated platelet destruction caused by drug-induced antibodies [2]. In 1985, the US Food and Drug Administration approved amiodarone for prophylaxis and treatment of potentially fatal ventricular arrhythmias [3-4]. In practice, it is widely used for the management of supraventricular tachyarrhythmia, especially atrial fibrillation/atrial flutter, prevention, cardiac arrest from refractory ventricular arrhythmias, treatment of postoperative tachyarrhythmia, and as an adjunct to an implantable defibrillator [4]. Physicians are familiar with the side effects of amiodarone, including hypothyroidism, interstitial pneumonitis, and hepatotoxicity [3]. An idiosyncratic reaction, such as amiodarone-induced immune thrombocytopenia (AITP), is a rare hematologic complication and has been described only a few times [5-8]. Bone marrow granulomas resulting from long-term amiodarone use is a non-immune-mediated cause of thrombocytopenia that is usually accompanied by other cytopenias [9-10].

## Case presentation

**Figure 1:**
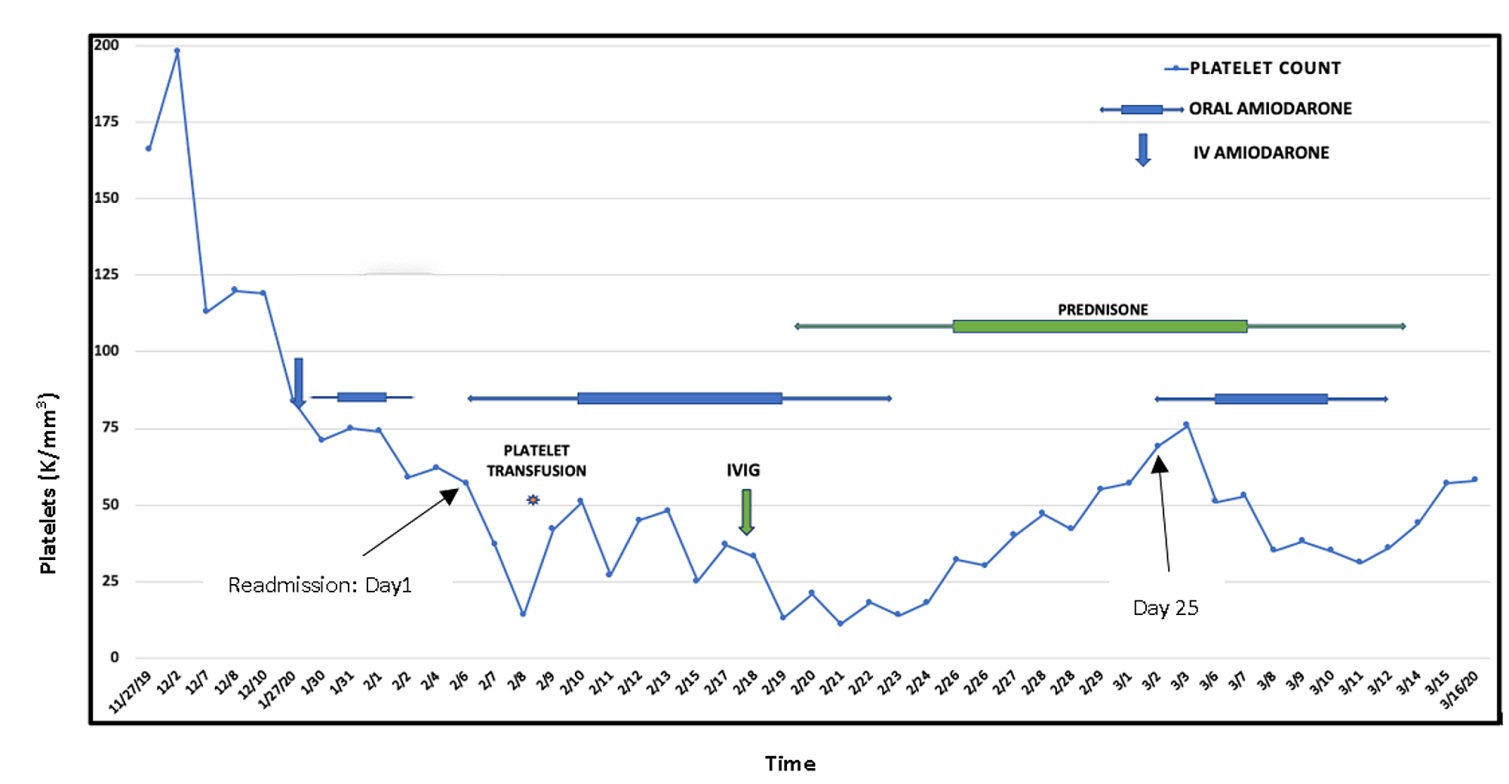
Course of immune thrombocytopenia with amiodarone IV: intravenous; IVIG: intravenous immunoglobulin

A 72-year-old male was admitted for hyperkalemia and symptomatic bradycardia after a missed hemodialysis session due to the occlusion of his arteriovenous fistula. His past medical history was significant for type 2 diabetes mellitus, hypertension, atrial fibrillation, coronary artery disease, congestive heart failure, end-stage renal disease secondary to diabetic nephropathy, and diabetic peripheral neuropathy. His home medications included aspirin, insulin, gabapentin, carvedilol, lisinopril, amiodarone, and warfarin. He was diagnosed with Enterobacter bacteremia and started on cefepime. Shortly before this, he was admitted for five days for atrial fibrillation with rapid ventricular rate and treated with intravenous (IV) amiodarone loading followed by an oral maintenance dose. He was discharged three days before the aforementioned admission. On Day 2, the laboratory studies revealed worsening thrombocytopenia with a platelet count of 37 K/mm^3^ (compared to 62 K/mm^3^ upon his previous discharge) which was presumed to be in the context of sepsis (Figure 1). Amiodarone was restarted for atrial fibrillation, and anticoagulation was held due to thrombocytopenia. On Day 3, a blood culture showed no growth, and all signs of sepsis had resolved. Antibiotics were discontinued. Yet, the platelet count went down to 14 K/mm^3^, at which point one unit of platelets was transfused. Fortunately, there was no evidence of bleeding. Response to transfusion was modest and temporary. Platelet count again decreased to 25 K/mm^3^, at which point the hematology team was consulted.

Pseudothrombocytopenia, alcohol abuse, nutritional deficiencies (Vitamin B12 deficiency, folate deficiency), human immunodeficiency virus (HIV), and hepatitis C virus (HCV) were ruled out. Heparin-induced thrombocytopenia antibody testing by enzyme-linked immunosorbent assay (ELISA) was negative (optical density (OD): 0.112). A review of peripheral blood smear showed decreased platelet count, but no platelet clumping or schistocytes and a few giant platelets were notable. The latter, in addition to mild reactive neutrophilia, pointed against the possibility of bone marrow suppression from sepsis. Giant platelets on peripheral blood smear and elevated immature platelet fraction at 20.1% pointed at rapid bone marrow turnover of platelets in the peripheral circulation. Abdominal imaging with CT scan ruled out splenomegaly; liver echotexture supported a functioning liver. Given that platelet count dropped below 20 K/mm^3^ despite resolution of sepsis, drug-induced immune thrombocytopenia secondary to amiodarone was entertained as a working diagnosis. He was on chronic hemodialysis for end-stage renal disease and was perceived to be at higher risk of bleeding due to functional coagulopathy from uremia [11]. Meanwhile, since AITP was a rare possibility, we were reluctant to stop amiodarone immediately for lack of better alternatives and risk of hemodynamic compromise. Hence, two doses of intravenous immunoglobulin (IVIG) 1 g/kg were administered on Day 11 followed by 1 mg/kg oral prednisone maintenance. Amiodarone was eventually discontinued on Day 17, considering AITP as a working diagnosis when the platelet nadir reached 14 K/mm^3^. Platelet recovery occurred in parallel to a peak of 76 K/mm^3^ on Day 26.

On Day 25, the patient experienced hemodynamic instability in the setting of tachyarrhythmias for which he was cautiously restarted on oral amiodarone due to lack of a better alternative. Two days later, platelet count started worsening and continued to do so until amiodarone was safely weaned off 11 days after restarting it. Thrombocytopenia started recovering the following day lending more credibility to earlier suspicion of an immune-mediated, amiodarone-dependent platelet destruction process. Unfortunately, the patient succumbed to a cardiac arrest before his platelet count could recover fully.

## Discussion

The incidence of AITP is unknown due to the rarity of this complication and the paucity of documented cases. DITP has been associated with an increased risk of in-hospital mortality [1]. A distinctive feature of one of the well-known examples of DITP, such as quinine-induced DITP, involves a platelet-reactive antibody that binds tightly to platelets only in the presence of quinidine, leading to immune destruction of platelets after five to seven days of continuous drug administration. In addition to the above, Aster and Bougie have summarized various mechanisms for DITP, some of which include hapten-dependent antibody (e.g., penicillin), autoantibodies which elicit an immune response in the absence of a drug (gold salt, procainamide), and immune complex formation (heparin) [2]. Weinberger et al. was the first to postulate delayed hypersensitivity as a mechanism for AITP by performing a lymphocyte stimulation test [6]. Monitoring a drop in platelet counts two weeks after starting amiodarone was recommended. Amiodarone-dependent antibodies causing platelet destruction cannot be identified using traditional serological methods owing to the water insolubility of the drug. This issue was elucidated by Sahud et al. who described a case series of three patients with AITP and employed elaborate laboratory techniques to demonstrate amiodarone-dependent antibodies specific for platelet glycoproteins GPIa/IIa and/or GPIIb/IIIa to explain thrombocytopenia in these patients [5]. 

Thrombocytopenia is one of the commonest reasons for inpatient hematology consultation. The differential diagnosis is long and sometimes multifactorial. Thrombocytopenia workup guidelines described by Arnold and Lim were useful to approach our case [12]. They have described a handy six-step approach to evaluate thrombocytopenia: 1) exclude thrombocytopenic emergencies, 2) peripheral blood smear examination, 3) consider clinical context, 4) degree, 5) timing, and lastly, 6) assess for bleeding/thrombosis. Table 1 highlights the salient features of drug-induced immune thrombocytopenia. In the case discussed here, firstly, a citrated platelet level ensured that the severely low platelet count was not a laboratory error. Fatal etiologies of severe (sometimes referred to as significant) thrombocytopenia, like disseminated intravascular coagulation (no overt bleeding, normal hemolysis markers, normal fibrinogen), thrombotic thrombocytopenic purpura (no schistocytes, no hemolysis), and heparin-induced thrombocytopenia (negative heparin-induced thrombocytopenia (HIT) ELISA screen, typical platelet nadir is 60 K/mm^3^ in HIT), were ruled out. DITP as a possible cause of severe thrombocytopenia was suspected, given the worsening thrombocytopenia to less than 20 K/mm^3^ despite prompt resolution of sepsis with the appropriate antibiotic, the timing of onset at Day 5 after starting amiodarone, and the presence of giant platelets on the blood smear. Severe sepsis can sometimes cause severe thrombocytopenia but that is often associated with severe leukopenia as a marker of severe bone marrow suppression. Amiodarone was listed as a possible cause of DITP in a commonly referred expanded online list of drugs and their level of association with thrombocytopenia [13]. Later during the clinical course, recurrence of thrombocytopenia with amiodarone introduction and improvement after drug discontinuation lent more support to the diagnosis. It must be pointed that drug rechallenge is not required to diagnose any DITP. Platelet reactive antibodies were not tested since amiodarone-dependent platelet-responsive antibodies are not detectable by conventional methods [5]. It was not feasible to apply the criteria laid out by George et al. to establish the causal relation of amiodarone to immune thrombocytopenia since the patient died prematurely before platelet counts could return to normal [14]. Regardless, the clinical picture pointed to AITP and there was no alternate explanation. Although platelet recovery may be relatively prompt after drug discontinuation, complete recovery may take a few weeks owing to the long half-life of amiodarone from its large volume of distribution [3, 7]. Antibodies can persist for years, and hence, reexposure of the drug even months to years later can elicit similar immune destruction of the platelets [15]. The offending medication must be added to the allergy list in order to maximize safety.

**Table 1:** Salient Features of Drug-induced Immune Thrombocytopenia (DITP) * sooner if previously sensitized with drug exposure ** drugs with longer half-lives may have delayed recovery

Salient Features of DITP
Acute life-threatening thrombocytopenia
Blood smear shows severe thrombocytopenia; giant platelets support the diagnosis
Degree of thrombocytopenia is usually severe: < 20 K/mm^3^
Timing of the onset is typically 5 - 10 days after drug exposure*
Thrombocytopenia recovers typically within days after drug discontinuation**

One of the most common indications for amiodarone is atrial fibrillation and this diagnosis requires anticoagulation for thromboembolic stroke prevention [16-17]. If a patient develops severe amiodarone-induced thrombocytopenia in this setting, anticoagulation may require interruption for several days due to the long half-life of amiodarone. This can increase the risk of thromboembolic stroke with serious clinical consequences.

The role of both high-dose steroids and IVIG in the management of DITP has not been well-established, unlike immune thrombocytopenia (ITP) where both of these drugs have a long track record of efficacy [18]. High-dose steroids can have serious side effects, including hypertension, poor glycemic control, gastric ulceration (thrombocytopenia compounds bleeding risk), edema, encephalopathy (especially, intravenous administration in elderly patients), anxiety, restlessness, insomnia which contribute to patient morbidity, and rarely, mortality during hospitalization. IVIG therapy, other than being expensive, carries the risk of serious allergic reactions. However, we believe that if there is a strong clinical suspicion of DITP and it is perceived that the patient is at risk of life-threatening hemorrhage from severe DITP despite discontinuation of the offending drug, then a trial of corticosteroids/IVIG can be justified. Platelet transfusion carries the risk of infection, allergic reaction, alloimmunization, fluid overload, and hence, its judicious use is advised. Platelet transfusion is recommended in a case of active bleeding with a platelet count under 50,000 [19].

## Conclusions

DITP can present as an acute or delayed-onset complication of amiodarone. Given its frequent use in clinical practice, it is crucial to diagnose this entity in a timely manner in order to prevent hemorrhagic complications.
